# Knowledge about cataract and associated factors among adults in Yirgalem town, Sidama National Regional State, southern Ethiopia, 2020: a community based cross sectional study design

**DOI:** 10.1186/s12886-021-01844-3

**Published:** 2021-02-10

**Authors:** Anteneh Fikrie, Yonatan G. Mariam, Elias Amaje, Henok Bekele

**Affiliations:** 1 School of Public Health, College of Health and Medical Sciences, Bule Hora University, PO. Box 144, Bule Hora, Ethiopia; 2 Public Health Department, Pharma College Hawassa Campus, P.O.B. 67, Hawassa, Ethiopia; 3 Malaria prevention, Control and Elimination Technical Advisory in South Nation Nationalities Peoples Regional State, Southern Ethiopia Hawassa, Ethiopia

**Keywords:** Cataract, Knowledge, Yirgalem town, Southern Ethiopia

## Abstract

**Background:**

Globally, at least 1 billion people have a vision impairment that could have been easily prevented or easily treated. Cataract is the leading preventable and most treatable causes of blindness and bilateral low vision among adults. Despite being the leading cause of preventable and most treatable blindness, the lack of knowledge about the disease and its option of treatment is still a major barrier in reducing the blindness owing to cataract in the developing countries particularly in Ethiopia. Hence, the aim of this study is to determine the level of knowledge about cataract and associated factors among adults in Yirgalem Town, Sidama National Regional State, Southern Ethiopia, 2020.

**Methods:**

A community-based cross-sectional study design was conducted among randomly selected 599 adult’s age 18 years and above from May 10–30, 2020. A multi-stage sampling technique was used to select the study participants. Data were collected using pre-tested and structured face-to-face interview questionnaires. The collected data were entered to Epi data version 3.1 and then exported to SPSS version 21 for analysis. Bi-variable and multivariable logistic regression was used to identify associated factors of knowledge about cataract. Adjusted Odds Ratio (AOR) together with 95% Confidence Interval (CI) was used to declare the statistical association between dependent and independent variables.

**Results:**

Of the total study participants, 379 (64.7%), [(95% CI: 60.7–68.6%)] of them had good knowledge about cataract. Age (≥40 years) [AOR = 2.29(95% CI 1.18–4.44)], Elementary school completed [AOR = 2.31(95% CI 1.30–4.10)], High school & above [AOR = 5.55(95% CI 2.81–10.89)], governmental and non-governmental employed [AOR = 5.62 (95% CI 2.78–11.38)], Merchant [AOR = 1.72(95% CI 1.03–2.88)], Positive Attitude [AOR = 3.85(95% CI 2.94–6.47)] were positively significantly associated with knowledge about cataract. Whereas, rural residence [AOR = 0.19 (95% CI: 0.12–0.31)] was negatively associated with knowledge about cataract.

**Conclusions:**

More than one third of the participants still had poor knowledge about cataract. This implies that health facilities should be engaged and raises the awareness of the community and empowers people about eye care needs.

**Supplementary Information:**

The online version contains supplementary material available at 10.1186/s12886-021-01844-3.

## Background

According to World Health Organization (WHO) cataract is clouding of the lens of the eye, which initially prevents clear vision and eventually progresses to blindness if left untreated [1]. It causes increased light sensitivity, decreased vision at night, seeing double images and leads to total blindness [2, 3]. Although the commonest causes of cataract are related to the aging process, occasionally children can be born with the condition, or a cataract may develop after eye injuries, inflammation, and some other eye diseases [4–6]. Evidence showed that women are at greater risk than men for developing cataracts [7]. It is the most avoidable condition if timely intervention is instituted; if not, it results in catastrophic complications that end up with irreversible blindness [3]. Consequently, its negative psycho-social economic impact is manifested at individual, family and community level [8].

Globally, at least 2.2 billion people have vision impairment. Among this 65.2 million people are suffered from with moderate or severe distance vision impairment or blindness due to cataract [1]. The proportion of blindness due to cataract among all eye diseases ranges from 5% in developed countries to 50% or more in low- and middle-income regions of western and eastern sub-Saharan Africa (5.1%) and South Asia [1, 9]. The prevalence of low vision and number of blind persons due to cataract in Ethiopia are estimated to be 3.7 and 1.6% respectively. It has been estimated that cases of cataracts will continue to rise [10]. This indicates that burden of eye disease in Ethiopia pose huge economic and social impacts on individuals, society and the nation at large.

Despite being the leading cause of treatable blindness, the lack of awareness shortage of trained eye health personnel, limited accessibility, high cost of treatment, and poor surgical outcomes are remains to be a snag in lessening the blindness owing to cataract, particularly in the developing countries, like Ethiopia. Knowledge about cataract alone is the most crucial facet for delaying the occurrence of cataract, to initiate regular eye checkup, and to institute timely intervention and this will [11]. This, in turn, reduces the burden of the disease. Furthermore, assessing knowledge regarding cataract is a precondition for designing health education and promotion programs.

Despite the presence of effective interventions to reduce the risk of acquiring vision impairment due to the cataract; such as health promotion, prevention, treatment and rehabilitation [1]. The lack of accessibility to health care and limited financial resources becomes prohibitive factors for patients with cataracts in developing countries [1, 9]. Moreover, the inadequate knowledge of the availability of services and disease by itself are also remains to be the hurdle of the problem [1, 11, 12]. Several previous studies finding also supported the presence of a gap in knowledge regarding cataract particularly in developing countries [11, 13, 14]. A study result revealed that attending higher level of education, higher family monthly income, previous eye examination and family history of cataract were identified as significant factors affecting the knowledge of cataract [12].

Despite the above problems, Ethiopia lacks accurate recent national estimates the knowledge of cataract and low vision. In addition to this, as to the best of our knowledge, a community based study on the assessment of the level of knowledge about cataract and associated factors among adult population was not done in our study area. Therefore, the result of this study will provide basic information for researchers, policymakers and resource allocators to plan health education and promotion programs allowing early cataract prevention and treatment options.

## Methods

### Study setting, design and period

A community-based cross-sectional study design was conducted at Yirgalem town from May 10–30, 2020. The town, which is one of the oldest towns found at Sidama National Regional State, Southern Ethiopia, is located about 47 km far from Hawassa City, the capital city of Sidama. According to the town health office report of 2019 the total population of the city is estimated to be 79,605, of which 39,166 are men whereas 40,439 are women. The urban population account for 48,605 and the rural accounts for 31,000.

### Sample size determination and sampling procedure

The sample size for this study was determined using single population proportion formula based on the following assumptions: Proportion of good knowledge about cataract (61.7%), which was conducted at Gondar town, Northwest Ethiopia [12], 95% Confidence interval [Z = 1.96), 5% margin of error, design effect of 1.5 and 10% non-response rate for the compensation of the potential non-responses. Then, the minimum calculated sample size became 599 Households. Sample size for second objective was computed by Epi info7 Statcalc version 7.1.4.0 software by the assumptions of, 95% level of confidence, power of 80%, the ratio of exposed to unexposed 1:1 and percent of outcome in unexposed group 38.98 and AOR of 2.02. The percent of outcome in unexposed group and AOR were taken from the study conducted in Northwest Ethiopia; the determinate variable was educational status of participants [12]. By substituting the above values in to software the estimated sample size was 282. By comparing the two sample size calculated, the first sample size was larger than the second as a result we took 599 as the final calculated sample size for the study.

A multi-stage sampling technique was employed to select the study participants. First, 4 kebeles were selected randomly using a lottery method from the total of 7 kebeles of Yirgalem town. Then, the sample size was allocated to the selected four kebeles based on their population proportion and simple random sampling technique was used to select the households. Finally one eligible adult was selected from each household using lottery method for the case where more than one adult was living in the house. All adult ≥18 years who reside in the study area for at least 6 months were included in the study. However, those adults who had psychiatric problems were excluded from the study.

### Operational definitions

#### Knowledge

The knowledge level of participants was determined based on 12 knowledge related questions, that consisted of six domains such as simple description, risk factors, symptoms, complications, treatment options and prevention strategies. Each item was equally weighted. Participants’ overall knowledge was categorized using the mean score, as good if the score was between 6 and 12 points and poor if the score was less than 6 points [12].

#### Attitude about the cataract

Participants’ overall score of attitude was assessed using 9 questions that consisted the supportive attitudes towards cataract prevention, Barriers related to patient attitude like (ability to manage routine work, cataract not mature, could see clearly with the other eye, busy with work), and management and finally who scored above the mean score among overall questions was categorized as positive or supportive attitude and negative if the score was less than the mean score [15].

### Data collection tools, procedures and quality control

The data were collected using a pretested structured questionnaire. The questions concerning knowledge about cataract with respect to definition, risk factors, symptoms, complications, and treatment option and prevention strategies were developed after literature review. The content of questionnaire with respect to clearness, easiness, included domains; reproducibility was validated by experts from Optometry department. The data were collected by trained 4 BSC optometrists through face to face questionnaire interview in the home to home visits. The questionnaire was primarily prepared in English version then translated in to local language; ‘Sidama Afoo’ then it was translated back into English version to check the consistence of the data. Two-day training was given to both data collectors and supervisors about the objective and methods of data collection. During the study supervisors were checked for completeness and correction. A pretest was conducted on 5% (*n* = 30) of the sample size to assess the quality of the tools at Abusto town. Based on the pre-test modifications was done as per necessary Data processing and analysis.

The collected data were checked for completeness and consistency, then entered into Epi data version 3.1 and exported to SPSS version 21 for analysis. The descriptive data were summarized using median and Interquartile range. Likewise, the data were presented using tables and charts. The model was checked using Hosmer- Lemeshow goodness of fit. Bi-variable and multivariable binary logistic regression was used to identify associated factors for knowledge about cataract. Variables with a *p*-value < 0.25 during the bi-variable logistic regression were further entered in to the final model, multivariate logistic regression so as to control confounding factors. A reliability analysis of the questionnaires was checked and Cronbach’s alpha showed the questionnaire were passed the acceptable reliability number (α = 0.77). Finally, the out puts of the bi-variable and multivariable binary logistic regression were reported and statistical significant were declared by AOR together with 95% Confidence interval.

## Results

### Socio- demographic characteristics of respondents

Among the total of 599 adults age ≥ 18 years, 586 of them were completed the interview to make a response rate of 97.8%. The median (±IQR) age is 31 years (±13 years). More than half of the study participants, 328 (56.0%) were females and 383 (65.4%) were urban dwellers. More than three-fourth and two-third of the respondents, 454 (77.5%) and 400 (68.3%) were married and protestant religion followers respectively. Regarding the educational level of the respondents, 260 (44.4%) had completed primary education. Two hundred fifty-six (43.7%) were Farmer. Nearly one-third, 196 (33.4%) of the participants household monthly income were less than 27.58 USD (Table 1).

**Table 1:** Socio-demographic characteristics of adults in Yirgalem town at Sidama national regional state, Southern Ethiopia, 2020

Variables	Category	No.	(%)
Sex of respondent	Male	258	(44.0)
	Female	328	(56.0)
Age (years)	18–29	229	(39.1)
	30–39	261	(44.5)
	40–49	51	(8.7)
	50 & above	45	(7.7)
Residence	Urban	383	(65.4)
	Rural	203	(34.6)
Marital status	Single	98	(16.7)
	Married	454	(77.5)
	Divorced	30	(5.1)
	Widowed	4	(.7)
Religion	Orthodox	73	(12.5)
	Muslim	69	(11.8)
	Protestant	400	(68.3)
	Catholic	33	(5.6)
	Others	11	(1.9)
Educational status	No formal education	105	(17.9)
	Primary education	260	(44.4)
	Secondary education	153	(26.1)
	College and above	68	(11.6)
Occupational status	Employed	134	(22.9)
	Merchant	196	(33.4)
	Farmer	256	(43.7)
Household monthly income	≤ 27.58$	189	(32.3)
	27.61–87.80$	201	(34.3)
	> 87.80$	196	(33.4)

(1$ = 38.0602 ETB)

### Previous history of eye examination of the study participants

Out of the total of 586 respondents, 181 (30.9%) of them underwent previous ocular examination where, 73 (12.5%) examined for cataract and 117 (20.0%) had regular eye checkup. Out of the total respondents, 24 (4.1%) had previous cataract diagnosis. More than half, 14 (58.3%) of them were claims lack of money, waiting time for surgery and health facility is far away from their home (Table 2).

**Table 2:** Previous history of eye examination of adults in Yirgalem town at Sidama national regional state, Southern Ethiopia, 2020

Variables	Category	No.	(%)
Previous eye examination	No	405	(69.1)
	Yes	181	(30.9)
Regularity of eye checkup	No	469	(80.0)
	Yes	117	(20.0)
Last eye visit	No	471	(80.4)
	Yes	115	(19.6)
Examine for cataract	No	513	(87.5)
	Yes	73	(12.5)
Previous cataract diagnosis	No	562	(95.9)
	Yes	24	(4.1)

### Participants’ attitude towards cataract

**Fig. 1:**
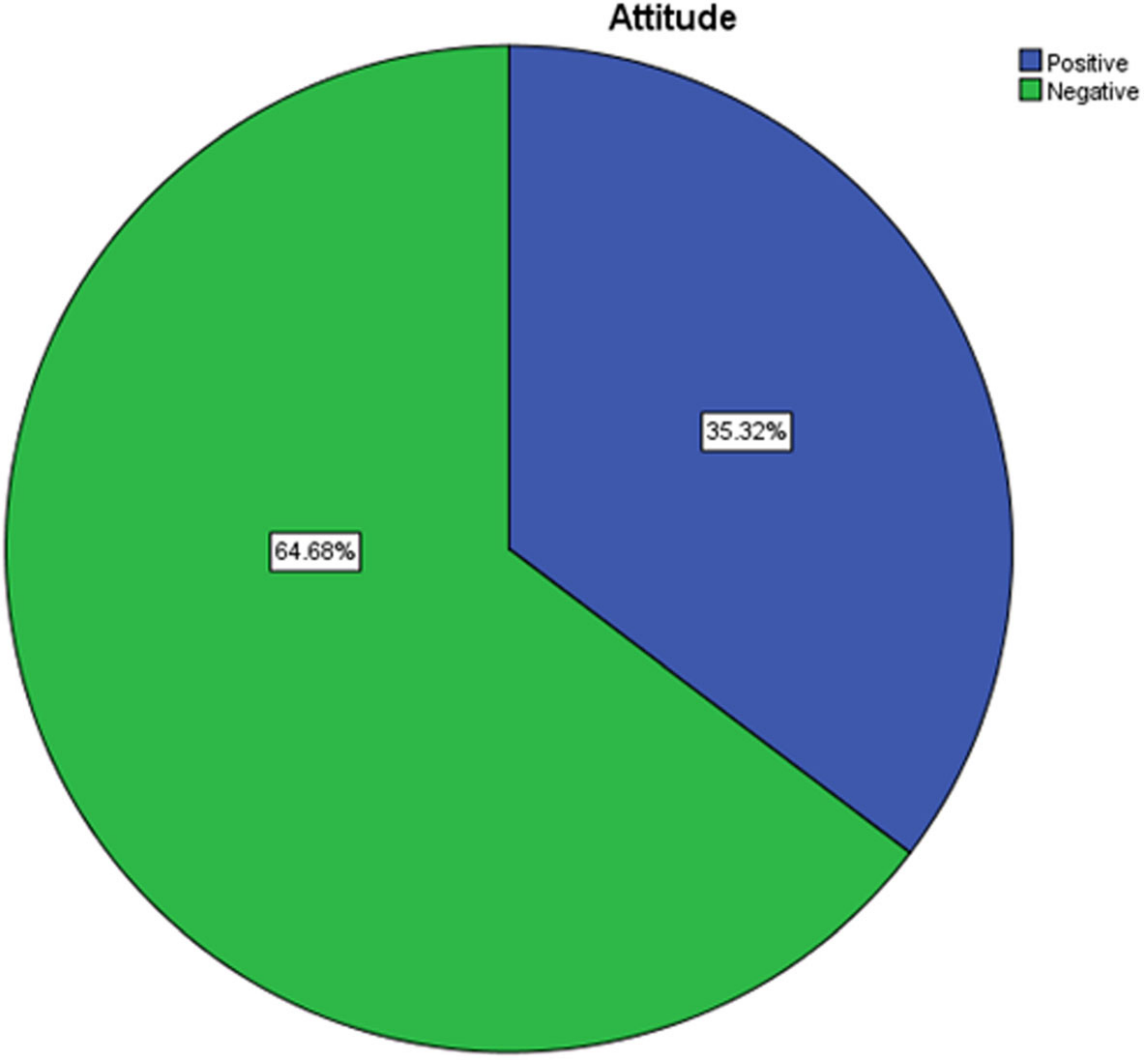
The attitude towards cataract among adults in Yirgalem town at Sidama national regional state, Southern Ethiopia, 2020

The mean (±SD) score of the overall participant’s attitude was 3.0 (±0.99). Regarding the attitude, more than one-third, 205 (35.0%) of the participants were afraid to undergo a cataract surgery while, 265 (45.2%) worried about the cost to incur for the cataract surgery and 257 (43.9%) had fear to the operation will lead to lose eye sight further more. Nearly half of the respondents, 311 (53.1%) believe that they could manage their work with one eye. On the other hand, half of the participants, 294 (50.2%) thought that operation will make them away from routine work for long time (Table 3). Regarding the participant’s attitude, as below figure shows, 207 (35.3%) had positive attitude towards cataract (Fig. 1).

**Table 3:** The participants’ attitude towards cataract among adults in Yirgalem town at Sidama national regional state, Southern Ethiopia, 2020

Variables	Disagree		Neutral		Agree	
	No.	(%)	No.	(%)	No.	(%)
Afraid to undergo a cataract surgery	261	(44.5)	120	(20.5)	205	(35.0)
Worried about the cost to incur for the cataract surgery	235	(40.1)	86	(14.7)	265	(45.2)
Afraid that the operation will lead to lose eye sight	243	(41.5)	86	(14.7)	257	(43.9)
Believe that could manage my work with one eye	216	(36.9)	76	(13.0)	294	(50.2)
Afraid that operation will make away from routine work for long time	213	(36.3)	62	(10.6)	311	(53.1)
Believe that poor eye vision is natural process and no need to intervene	199	(34.0)	93	(15.9)	294	(50.2)
Worried that their partner, children and relatives will have to suffer due to eye surgery	227	(38.7)	89	(15.2)	270	(46.1)
Afraid that they will have to wait long periods of time in the waiting list to do the operation	206	(35.2)	80	(13.7)	300	(51.2)
Believe that they were too old to undergo an eye surgery	199	(34.0)	89	(15.2)	298	(50.9)

### Participants’ knowledge about cataract

In our study more than three-quarter, 506 (86.3%) of the participants had heard of cataract. The mean (±SD) knowledge score point was 6.65 ± 3.58 points. More than three-in-five of the participants mentioned at least one symptoms of cataract 323 (63.8). More than three-fourth, 272 (76.4%) of the study participants main source of information was health professionals followed by family, 45 (12.6%), Friends or Neighbor 23 (6.5%) and media 16 (4.5%). Regarding risk factors of cataract, 392 (77.5%) of the participants identified older age as a principal. On the other hand two-third, 335 (66.2%) of the participants were correctly replied about the possibility of prevention of risk factor of cataract.

**Fig. 2:**
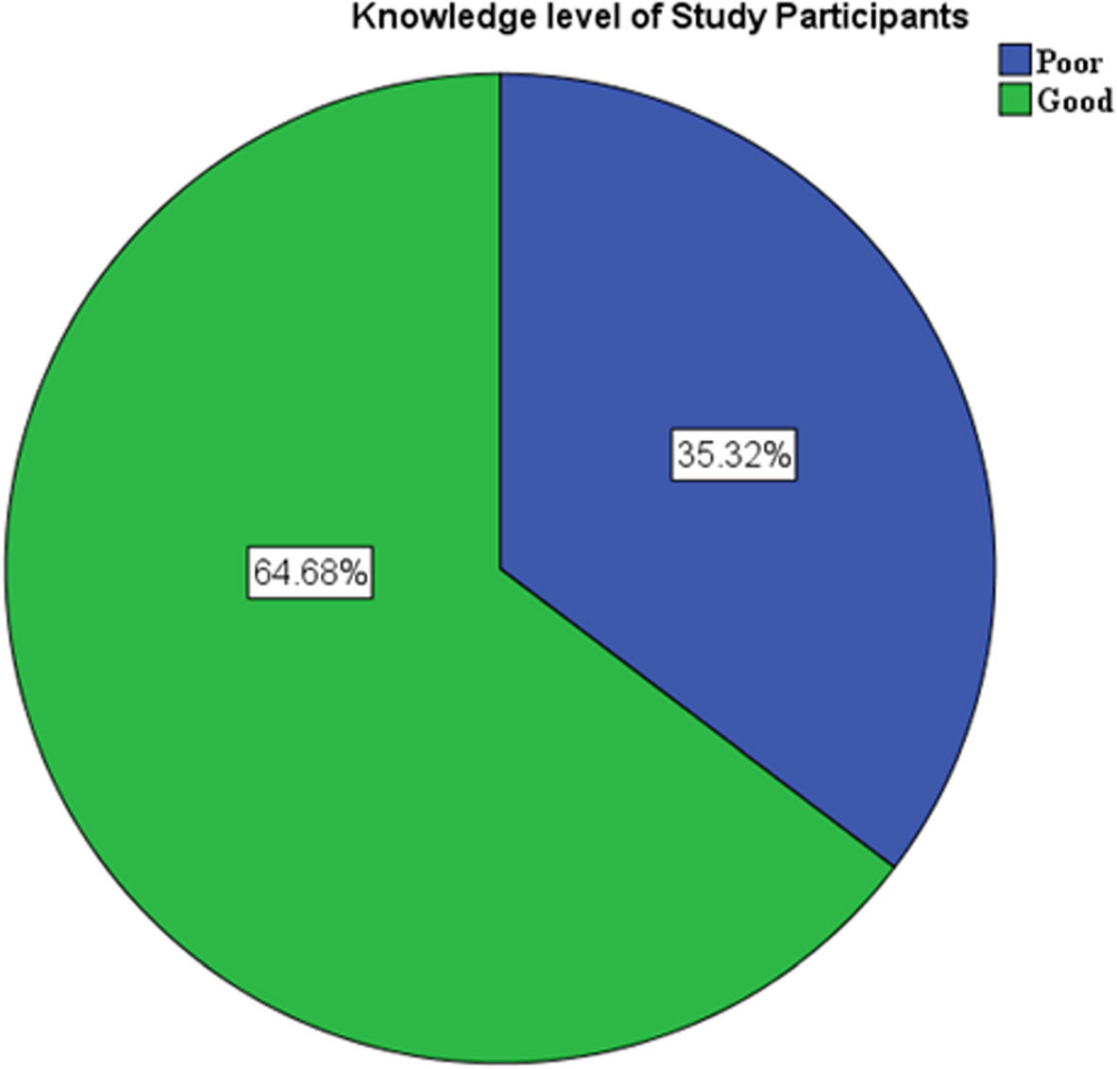
The overall knowledge about cataract of adults in Yirgalem town, Sidama national regional state, southern Ethiopia, 2020

The vast majority, 506 (86.3%) of participants were mentioned at least one prevention of risk factor of cataract, where 463 (91.5%) and 377 (74.5%) of participants mentioned Vitamin supplementation and quitting cigarette smoking as a prevention mechanism of the risk factors for cataract. Half, 260 (51.4%) of the participants were aware of that government hospitals provide treatment for cataract. However, less than 50 % of the participants were know that cataract surgery be done free of charge 212(41.9%). Nearly 60 %, 299 (59.1%) of participants comprehended that the reversibility of vision after cataract treatment is possible. Whereas, 314 (62.1) of the participants were perceived that the worst effect will occur unless cataract is treated. Fifty percent, 254 (50.2) of the participants answered that the best treatment option for cataract is surgery (Table 4). According to the finding of our study, nearly two-in-three, 379 (64.7%) [(95% CI: 60.7–68.6%)] of the participants had good knowledge about cataract (Fig. 2).

**Table 4:** Participants’ knowledge about cataract of adults in Yirgalem town at Sidama national regional state, Southern Ethiopia, 2020

Variables	Responses	
	Yes n (%)	No n (%)
Have you ever heard about cataract	506 (86.3)	80 (13.7)
Older age risk factor of cataract	392 (77.5)	150 (29.6)
Trauma risk factor of cataract	150 (29.6)	392 (77.5)
Prevention of risk factors is possible	335 (66.2)	171 (33.8)
Mechanism of prevention mentioned at least one	506 (56.3)	80 (13.7)
Symptoms of cataract	323 (63.8	183 (36.2)
Worst effect will occur unless treated cataract	314 (62.1)	192 (37.9)
Surgery is the best treatment option.	254 (50.2	252 (49.8)
Reversibility of vision after treatment is possible	299 (59.1)	207 (40.9)
Government hospitals provide treatment for cataract	260 (51.4)	246 (48.6)
It necessary to implant a lens in cataract surgery	351 (69.4)	155 (30.6)
Cataract surgery be done free of charge	212 (41.9)	294 (58.1)

### Factors associated with knowledge about cataract

After controlling the potential confounders by the multivariate analysis: Rural residence [AOR = 0.19 (95% CI:0.12–.31)], Age (≥40 years) [AOR = 2.29(95% CI 1.18–4.44)], Elementary school completed [AOR = 2.31(95% CI 1.30–4.10)], High school & above [AOR = 5.55(95% CI 2.81–10.89)], Both government and private employed [AOR = 5.62 (95% CI 2.78–11.38)], Merchant [AOR = 1.72(95% CI 1.03–2.88)], Positive Attitude [AOR = 3.85(95% CI 2.94–6.47)] were significantly associated with knowledge about cataract. Accordingly, the odds of good knowledge were 81% reduced among study participants from rural residence as compared to the urban resident [AOR = 0.19 (95% CI: 0.12–0.31)]. Similarly, adults aged Age (≥40 years) were 2 times more likely to have good knowledge about cataract as compared with younger age [AOR = 2.29(95% CI 1.18–4.44)].

Those participants with an educational level of primary school completed had 2.3 times more likely to have good knowledge than those who do not have formal education [AOR = 2.31(95% CI 1.30–4.10)]. Similarly, those participants with educational level of high school and above were 5.5 times more likely of having a good knowledge as compared to those who do not have formal education [AOR = 5.55(95% CI 2.81–10.89)]. The study respondents who were employed and merchant were 5.6 [AOR = 5.62 (95% CI 2.78–11.38)], and 1.7 [AOR = 1.72(95% CI 1.03–2.88)] times more likelihood of having a good knowledge about cataract than farmers respectively. Likewise, study participants who have positive attitude about cataract were nearly 4 times more likely of having good knowledge as compared to those study participants who have negative attitude [AOR = 3.85(95% CI 2.94–6.47)] (Table 5).

**Table 5:** Factors associated with knowledge about cataract of adults in Yirgalem town at Sidama national regional state, southern Ethiopia, 2020

Variables	Knowledge of cataract				COR (95%CI)	AOR (95%CI)
	Good		Poor			
	No.	(%)	No.	(%)		
Sex of respondent						
Male	172	(66.7)	86	(32.3)	1	–
Female	207	(63.1)	212	(36.9)	0.85 (0.60–1.20)	–
Residence						
Urban	284	(74.2)	99	(25.8)	1	1
Rural	95	(46.8)	108	(53.2)	0.30 (0.21–0.41)	0.19 (0.12–.31)***
Marital status						
Married	292	64.3	162	35.7	1.60 (0.79–3.22)	
Single	69	70.4	29	29.6	2.11 (0.94–4.71)	
Others^@^	18	52.9	16	47.1	1	1
Age (years)						
18–29	129	(56.3)	100	(43.7)	1	1
30–39	175	(67.0)	86	(33.0)	1.57 (1.09–2.27)	1.15 (0.71–1.85)
≥ 40	75	(78.1)	21	(21.9)	2.76 (1.59–4.79)	2.29 (1.18–4.44)*
Educational status						
No formal education	45	(42.9)	60	(57.1)	1	1
Elementary school	151	(58.1)	109	(41.9)	1.84 (1.16–2.92)	2.31 (1.30–4.10)**
High school & above	183	(82.8)	38	(17.2)	6.42 (3.81–10.81)	5.55 (2.81–10.89)***
Household monthly income						
≤ 1000 ETB	115	(60.8)	74	(39.2)	1	1
1001–2575 ETB	129	(64.2)	72	(35.8)	1.15 (0.76–1.73)	1.16 (0.68–1.97)
≥ 2575 ETB	135	(69.9)	61	(31.1)	1.42 (0.93–2.16)	1.15 (0.67–1.97)
Occupation						
Employed	118	(88.1)	16	(11.9)	7.49 (4.20–13.33)	5.62 (2.78–11.38)***
Merchant	134	(68.4)	62	(31.6)	2.19 (1.48–3.23)	1.72 (1.03–2.88)*
Farmer	127	(49.6)	129	(51.4)	1	1
Previous eye examination						
Yes	161	(89.0)	20	(11.0)	6.90 (4.17–11.43)	3.08 (1.00–9.38)
No	218	(53.8)	187	(56.2)	1	1
Family history of cataract						
Yes	141	(90.4)	15	(9.6)	7.58 (4.30–13.34)	2.12 (0.61–7.36)
No	238	(55.3)	192	(44.7)	1	1
Attitude level						
Positive	178	(86.0)	129	(14.0)	5.43 (3.49–8.45)	3.85 (2.94–6.47)***
Negative	201	(53.0)	178	(47.0)	1	1

NB; ^@^ Divorced, Widowed, *** *P*-value < 0.001,** *P*-value < 0.01,,* *P*-value < 0.05,

## Discussions

This study result revealed that the overall good knowledge about cataract among the study participants was 379 (64.7%) [(95% CI: 60.7–68.6%)]. This finding was consistent with a study conducted in Gondar town, northwest Ethiopia, 61.7% [12]. However, this study finding was lower as compared with other studies; 74.6% in Nepal [16], 87% in rural Peru [17], 74% Iran [18], and 70.9% China [6]. This difference might be due to the variation of the technological advancement of the country, implementation of the health care strategy and the better socio-economic development of the communities. However, this study finding was higher than the study report of Southern Indian 15% [11]. This discrepancy might be the reason for existed difference in socio economic and demographic factors between the study participants. In addition to this the reason for discrepancy might be the difference in target population and study setting.

The study result showed that, the odds of good knowledge were 81% reduced among study participants from rural residence as compared to the urban resident. This study finding is similar with other studies conducted abroad, central rural India [19] and Ontario Canada [15]. This might due to the fact that people living in urban have access to different sources of information regarding the issues in addition to this people living in urban areas have better exposures to media and eye care professionals that increase access to health education and knowledge. Moreover, the rural populations also face greater barriers to accessing eye care due to distances to travel and poor road quality, among other factors.

Adults 40 years and above were two times more likely to have good knowledge as compared with younger aged group. This is consistent with the study reports from Ontario Canada [15], surveys in China [20, 21]. This might due to the nature of the disease in which the prevalence increases sharply with age and that may lead to support growing demands for cataract care in our aging population.

This study result showed that, participants with an educational level of completed primary and high school and above were 2.3 and 5.5 times more likely of having a good knowledge as compared to those who do not have formal education. This study finding is similar with a study report from Gondar town, northwest Ethiopia [12], Hilly Region of Nepal [16] and Southwest Nigeria [22]. This might explain that individuals with higher educational level would read more and use social media so they would become more knowledgeable.

The study respondent’s occupational status being employed and merchant had good knowledge about cataract as compared with farmers. This study finding was agreed with the previous study report from Eastern Nepal [13] and China [23]. This might explain that individuals with occupation makes to be exposed to different media and favors access to health facilities with better economic level and also would have an opportunity to access eye care service. This implies that farmers are more likely to have poor exposures to media and eye care professionals that increase access to health education and knowledge. Likewise, the nature of the occupation could also facilitate the interaction between different individuals with varied skills, so this might also let them to have information, skills and knowledge sharing mechanisms.

Likewise, study participants who have positive attitude about cataract were nearly 4 times more likely of having good knowledge as compared to those who have negative attitude. This might be due to the fact that, positive attitude of the study population towards cataract can increase the populations awareness regarding the causes, prevention and management for cataract. Moreover, attitudes can guide encoding information, attention and also helps to maintain organized, meaningful, and stable behaviors.

The main strength of this study is that it lies on the large sample size with high response rate. This study has its own limitation on response biases, recall biased on socio-demographic factors like exact age, which may affect the result of this study. The factors do not establish temporal relationship due to the nature of the cross-sectional study design.

## Conclusion

This study shows that more than one third of the participants still had poor knowledge about cataract. Majority of the participants had heard of cataract. The rural dwellers, the respondents 40 years and older, at least elementary education and above, merchant and employed had significant association with good knowledge about cataract. Organizing different health education programs addressing on risk factors like attitudes problems and rural communities who had few accesses to mass-media and different prevention methods to delay occurrence of the disease is essential to improve the knowledge regarding the cataract.

## Supplementary Information

**Additional file 1.**

## Data Availability

For those who are interested; the datasets of this study could be accessed from the corresponding author on reasonable request.

## Abbreviations

AOR: Adjusted Odds Ratio
COR: Crude Odds Ratio
CI: Confidence Interval
CSA: Central Statistical Agency
EDHS: Ethiopia Demographic and Health Survey
FMOH: Federal Ministry of Health
HSTP: Health Sector Transformation Plan
RDT: Rapid Diagnostic Test
SDGs: Sustainable Development Goals
SPSS: Statistical Package for Social Science
WHO: World Health Organization
