# Supplementary material for: Knowledge about cataract and associated factors among adults in Yirgalem town, Sidama National Regional State, southern Ethiopia, 2020: a community based cross sectional study design
Source: BMC Ophthalmol. 2021 Feb 10;21:79. doi: 10.1186/s12886-021-01844-3 (PMC7877085; doi:10.1186/s12886-021-01844-3)
Supplement: Supplementary file 1 — Additional file 1. [file 12886_2021_1844_MOESM1_ESM.docx]

**Knowledge about Cataract and Associated Factors among Adults in Yirgalem Town, Sidama National Regional State, Southern Ethiopia, 2020: A Community Based Cross Sectional Study Design**

Anteneh Fikrie^1, 2*^, Yonatan G/Mariam ^2^, Elias Amaje^1^, Henok Bekele^2,3^

1. School of Public Health, College of Health and Medical Sciences, Bule Hora University, PO. Box 144, Ethiopia.

2. Public Health Department, Pharma College Hawassa Campus, Hawassa, Ethiopia, P.O.B. 67 Ethiopia

3. Malaria prevention, control and elimination technical advisory in South Nation Nationalities peoples regional state, Southern Ethiopia.

***Correspondent Author**: Anteneh Fikrie, E-mail: [antenehfikrie3@gmail.com](mailto:antenehfikrie3@gmail.com)

Phone: +251922465129

ENGLISH VERSION QUESTIONNAIRE

**PART I: SOCIO-DEMOGRAPHIC CHARACTERISTICS**

| **No** | **Questions** | **Coding Categories** | **Skip** |
| --- | --- | --- | --- |
|  | Sex of respondent | 1. Male 2. Female |  |
|  | Age (Incomplete years) | -___________________ |  |
|  | Address | 1. Urban 2. Rural |  |
|  | Marital status | 1. Single 2. Married  3. Divorced 4. Widowed |  |
|  | Religion | 1. Protestant 2. Orthodox  3. Catholic 4. Muslim  5. Other specify ____________ |  |
|  | Educational status | 1. No formal education 2. Elementary completed 3. Secondary Completed 4. College & above |  |
|  | Occupational status | 1. House wife 2. Farmer 3. Government employee 4. Private employee 5. Others |  |
|  | Average Household monthly income? | ______________ birrs |  |

**PART 2: QUESTIONS RELATED TO THE KNOWLEDGE OF PARTICIPANTS ABOUT CATARACT**

| **No** | **Questions related to the Knowledge** | **Coding Categories** | **Skip** |
| --- | --- | --- | --- |
|  | Have you ever heard about cataract? | 0. No 1. Yes |  |
|  | If Yes, what is your source of the information? | 1. Health professionals 2. Media 3. Family 4. Friends/ Neighbor 5. Others |  |
|  | Can age be a risk factor for cataract? | 0. No 1.Yes |  |
|  | Can trauma be a risk factor for cataract? | 0. No 1. Yes |  |
|  | Prevention of risk factors is possible? | 0. No 1.Yes |  |
|  | What is the mechanism of prevention of cataract? | 1. Minimize alcohol use 2. Quit Cigarette smoking 3. Normal body weight 4. Vitamin supplement 5. Nutritional support 6. Others ________ |  |
|  | What is the best treatment option for cataract? | 1. Wearing glasses 2. Surgery 3. Drugs 4. Laser treatment 5. Vitamin supplement 6. Nutrition treatment 7. Visiting ophthalmologist 8. Traditional herbal medicine 9. Others |  |
|  | The best treatment option for cataract is surgery? | 0. No 1. Yes |  |
|  | Do government hospitals provide treatment for cataract? | 0. No 1. Yes |  |
|  | Is it necessary to implant a lens in cataract surgery? | 0. No 1. Yes |  |
|  | Can cataract surgery be done free of charge? | 0. No 1. Yes |  |
|  | Symptoms of cataract | 0. No 1. Yes |  |
|  | Signs/symptoms of cataract mentioned | 1. Images look blurred & hazy 2. Less colorful 3. Fevered/Headache 4. Vomiting 5. Others |  |
|  | Worst effect will occur unless treated cataract | 0. No 1. Yes |  |
|  | Reversibility of vision after treatment is possible | 0. No 1. Yes |  |

**PART 3: QUESTIONS RELATED TO THE ATTITUDE OF PARTICIPANTS TOWARDS CATARACT**

|  | | Strongly Disagree (1) | Disagree (2) | Neutral (3) | Agree (4) | Strongly Agree (5) |
| --- | --- | --- | --- | --- | --- | --- |
|  | I am afraid to undergo a cataract surgery |  |  |  |  |  |
|  | I am worried about the cost I have to incur for the cataract surgery |  |  |  |  |  |
|  | I am afraid that the operation will lead to lose my eye sight further more |  |  |  |  |  |
|  | I believe that I could manage my work with one eye |  |  |  |  |  |
|  | I am afraid that operation will make me away from my daily routine work for long time |  |  |  |  |  |
|  | I believe that my poor eye vision is natural process and no need to intervene |  |  |  |  |  |
|  | I am worried that my partner, children and relatives will have to suffer due to eye surgery |  |  |  |  |  |
|  | I am afraid that I will have to wait long periods of time in the waiting list to do the operation |  |  |  |  |  |
|  | I believe that I am too old to undergo an eye surgery |  |  |  |  |  |

**PART 4: QUESTIONS RELATED TO PREVIOUS HISTORY OF PARTICIPANTS EYE EXAMINATION**

| **No** | **Questions** | **Coding Categories** | **Skip** |
| --- | --- | --- | --- |
|  | Do you have cataract | 0. No  1. Yes |  |
|  | Did you examine for cataract? | 0. No  1. Yes |  |
|  | Previous eye examination | 0. No  1. Yes |  |
|  | Regularity of eye checkup | 0. No  1. Yes |  |
|  | Last eye visit | 0. No  1. Yes |  |
|  | Previous cataract diagnosis | 0. No  1. Yes |  |
|  | Why don’t people get operated early? | 1. Lack of money 2. Waiting time for surgery 3. Health facility is far away 4. Apprehensive of complications 5. Vision is not lost totally 6. Fear of surgical procedure 7. Others _________________ |  |
|  | Any Other barrier for utilization of the service | 1. Inadequate resources 2. unavailability of trained staff, 3. Personnel turnover in government 4. Use of traditional preventive approaches 5. Others _________________ |  |
|  | Family history of cataract | 0. No 1. Yes |  |
|  | Is it possible for you to find transport to go to hospital? | 0. No 1. Yes |  |
|  | Do you expect assistance from your family members/children/relatives? | 0. No 1. Yes |  |
|  | Will you able to pay for drugs? | 0. No 1. Yes |  |

**Thank you very much for your voluntarily participation on the study!!!**
